# Attracting medical school graduates to residency programs in remotely located hospitals: the challenge lies beyond financial incentives

**DOI:** 10.1186/s13584-024-00629-5

**Published:** 2024-08-26

**Authors:** Shalev Fried, Ofira Zloto, Avia Doron, Zeev Feldman, Alexey Belinsky, Gad Segal, Yael Frenkel-Nir, Arnon Afek

**Affiliations:** 1 Faculty of Medicine, Tel Aviv University, Tel Aviv, Israel; 2 The Goldschleger Eye Institute, Sheba Medical Center, Tel-HaShomer, Israel; 3 Department of Obstetrics and Gynecology, Sheba Medical Center, Tel-HaShomer, Israel; 4 Department of Neurosurgery, Sheba Medical Center, Tel-HaShomer, Israel; 5 Division of Health Workforce and Infrastructure Forecast, Ministry of Health, Jerusalem, Israel; 6 Education Authority, Sheba Medical Center, Tel-HaShomer, Israel; 7 General Management, Sheba Medical Center, Tel-HaShomer, Israel

**Keywords:** Residency, Recruitment, Periphery, Remote location, Financial incentive, Monetary grant, Students

## Abstract

**Background:**

Recruitment to residency programs in hospitals located in other than major hubs (“remotely located”) is a challenge in many countries. In 2011, the Israeli Ministry of Health launched a 10-year financial incentive to encourage physicians to enroll in residency programs in such hospitals. Nearly 1 billion New Israeli Shekels (260 million US$) were invested in that program which had only limited success. As a new physician association’s collective agreement is impending, we aimed to measure the effectiveness of selected incentives in attracting medical school graduates to residencies in remotely located hospitals.

**Methods:**

This study included Israeli medical students in their final year of medical school. We used an online questionnaire with multiple-choice demographic questions and a 5-point Likert scale to gauge the effect of various incentives on their preference for residency location.

**Results:**

Between July and November 2022, 522 students responded (405 studied in Israeli medical schools [out of 705 students] and 117 in foreign medical schools [out of 1936 students]). Forty-two percent had at least one clerkship in a remotely located hospital, and 24% had included at least one remotely located hospital among their top five choices for internship. Only 13% reported that they prefer a residency program in those institutions. The incentive selected by students as most persuasive was government assistance in acceptance to and financial support for a fellowship abroad, followed by a financial grant and fewer on-call hours. Only 7% of the students indicated that no incentive would influence them to choose a remotely located hospital for their residency training. Medical education in a remotely located university and the choice of at least one remotely located hospital among the top five choices for internship were significantly associated with positive incentive receptivity, whereas male sex and older age were associated with negative receptivity.

**Conclusion:**

This study on the attitudes of Israeli medical school graduates toward incentives aimed at attracting them to residencies in remotely located institutions revealed that career development opportunities and assistance in obtaining fellowships might influence their choice.

**Supplementary Information:**

The online version contains supplementary material available at 10.1186/s13584-024-00629-5.

## Introduction

Geographical maldistribution of medical doctors is a challenge encountered in many countries; with physicians tending to prefer urban areas to those in remote locations due to concerns about their career development, work-life balance, and lifestyle [1, 2]. Various terms are used to describe underserved geographical regions, among them "rural areas" and "remote areas" internationally, or "peripheral areas" in Israel. Major cities are often seen as being more attractive to the younger generation of physicians due to the cultural, social, and professional advantages they offer [3]. Furthermore, healthcare positions in major cities are often perceived as being more prestigious, as well as providing better employment prospects and educational advancements, and as having better access to private practice [4]. This imbalance may contribute to significant inequality in healthcare outcomes between populations in remote and central areas, and, indeed, such differences have been reported in both developed and developing countries worldwide [4, 5]. While physician density has increased in most Organization for Economic Co-operation and Development (OECD) countries in recent decades, gaps in physician density among different regions remain a significant problem in all of them [2].

This issue has been a matter of concern for policymakers over the last decades, and various strategies and interventions have been employed with the intention of influencing physicians' choice to enroll to residency programs and practice in the remote locations. The main approaches have included incentive programs, selection of future physicians from those areas, coercion, regulations, and educational programs [6]. Previous investigations on what may attract medical students to remote locations have reported varying effects of financial incentives, although many studies have observed that financial incentives alone are usually insufficient [3, 4, 6–8]. For example, despite government interventions in Ontario, Canada, including the funding of a rural-focused medical school, financial incentives, and the introduction of attractive contracts for physicians, the shortage of healthcare professionals in those communities persisted [9]. Similarly, in the United States, various programs have been implemented to address physician shortages in rural areas, with most focusing upon recruiting new physicians rather than retaining those already practicing there [10]. In addition, a recent study from Germany identified several interventions that could influence students' decisions to practice in rural areas, among them higher compensation, financial grants, funding fellowships, infrastructure improvements, promoting cultural and medical diversity, and providing high-quality internships in rural settings [11].

Medical students' considerations regarding specialty selection have altered over the years. Unlike medical students in the past, contemporary medical students more strongly emphasize their need for work–life balance, stable working hours, learning opportunities, and independence in patient care than they do a higher income and prestige [12–15]. Furthermore, prestige, salary, and career prospects were often perceived as important by students seeking surgical residencies, as opposed to students seeking primary care or internal medicine, who perceived family, residency location, and highly ranked match as more important and were less influenced by career-related factors, such as income, workload, or promotion [16–20]. Those variables were also common among practicing physicians' favoring of different medical specialties. For example, internal medicine physicians with a subspeciality evaluated career-related criteria as more important than personal criteria and gave more weight to achieving a leadership position as a main goal compared to general internal medicine physicians [13].

### The Israeli case

Israel is unique in that although it is small in size (22K km^2^), there are significant disparities in healthcare between locations remote from central hubs and which are geographically situated mostly in the southernmost and northernmost areas of the country, and less in its central region [21]. In Israel, the geographical term "periphery" was also defined by government decision number 1060 as representing "areas of national priority" [22]. This definition comprises the socio-economic index and the "peripherality index" of the locality. The "peripherality index" includes the proximity of the local authority to the Tel-Aviv district (central), the proximity of the local authority to population concentrations, and the size of the population. One inequity example is the uneven distribution of healthcare professionals between the central and remote areas of the country, with the average number of medical doctors per capita being 5.5 in the central region compared to 2.7 in the northern and southern areas between 2019 and 2021 [23]. In 2011, the Israeli Ministry of Health launched a comprehensive program as part of the physicians' collective agreement and in cooperation with the Israeli Medical Association. The aim of the program was to encourage physicians to practice in remote locations or to choose a residency in fields lacking sufficient numbers of specialists. Remotely located hospitals were defined by a committee representing non-central hospitals in areas with comparatively lower physician density based on those "areas of national priority" [22]. The program included financial grants (beginning with 300K–500K New Israeli Shekels [NIS], approximately 80K–130K US$), and higher salaries in remote locations. The financial grants were intended to be available for 10 years; however, the allocated budget was insufficient after 5 years. The program was adjusted during this time period and additional sums were made available [24], but it came to a close years before 2020. Beginning in 2015, grants were extended to specialist doctors, in addition to residents, based on the needs determined by the discretion of remotely located hospital managers. Furthermore, grants were awarded to doctors who began their specialization in remote areas only if they met additional criteria such as the residency field [25]. In the notification regarding the discontinuation of the program, the Ministry of Finance concluded that although the incentive program does encourage physicians to work in remote areas, it should be further evaluated to determine whether it meets the current system’s needs [26]. According to another government report, it is estimated that almost 2 billion NIS had been invested in the program between 2011 and 2019, of which 941 million NIS were allotted to attract residents to remote locations (including 281 million NIS as financial grants), and 1.05 billion NIS to attract residents to the clinical disciplines with shortage in the number of specialists (including 593 million NIS as financial grants) [27].

Despite the investment of these substantial sums, the probability of a young physician to select a residency program in a remotely located medical center had only increased from 7 to 12%, suggesting that this intervention was probably insufficient [27]. Furthermore, two of Israel's medical schools are located in remote areas of the country (Ben-Gurion University in Be'er Sheva in the Negev region and Bar-Ilan University in Safed in the Galilee region). Interventions to increase the number of students studying in those medical schools were apparently unsuccessful, and students who were already there reported not wanting to practice in remotely located areas following medical school because they perceived that doing so would compromise their professional development [28]. The Israeli government recently established the "Ilanot" program, which aimed to "…target and cultivate youth with close ties to the periphery as the next generation of physicians" [29]. This program provides 70 additional places beyond existing quotas for medical students in those two medical schools who were either born in or have strong connections to the Negev or the Galilee regions, and it is expected to expand the enrollment.

In a study by Ashkenazi et al. [6], Israeli residents mentioned several factors as important in their decision of residency location: the quality of the department, the attitude toward residents and the effect of their choice on their professional advancement. A more recent study showed that residents who chose residency programs in remotely located hospitals in Israel noted that shortages in personnel, technologies, and infrastructures, high bed occupancy rates, and the need to serve diverse population groups as being challenges that needed to be met there. They concluded that it is essential to address both their personal and their professional needs and expectations in order to attract and retain them in those remote areas [3]. Wasserstrum et al. [8] studied the effects of the financial grants to attract residents to remotely located hospitals between 2012 and 2014. Those authors reported that the medical students who chose a residency program in the remotely located areas gave more importance to the financial grants in their decision-making than those who chose residency program in centrally located medical centers. Another study exploring this incentive program evaluated 500 fifth-year medical students from a single university in Israel: approximately 30% of those students indicated that the monetary grants tempted them to consider a residency in the periphery of Israel [30]. Thus, despite the great effort and heavy monetary investment, the effect of those programs was only partial [27]. It is rather trivial to question whether those 1 billion NIS could have been invested more efficiently to strengthen the healthcare system in the periphery of Israel.

### Israeli medical training

In Israel, medical education consists of either a 6-year program (divided into 3 years of pre-clinical education and 3 years of clinical training) or a 4-year graduate program (divided into 2 years of pre-clinical education and 2 years of clinical training). Both are followed by a mandatory year of internship. Graduates of foreign medical schools must take a medical qualification examination and complete the same mandatory year of internship. The internship location for both Israeli and foreign medical school graduates is determined by the Israeli Ministry of Health by using a lottery system on the basis of the student's list of preferences. The residency length ranges from 3 to 7 years, depending upon the field of specialty. The application is made directly by the student and the admission to residency in the desired department is made directly by the institution without any governmental intervention or standardization of the number of residents in each department or medical field [31].

Nearly 60% of the physicians in Israel obtained their medical degree in a foreign country [31]. While the professional level of medical education in Israeli institutions is considered high**,** the quality of foreign medical schools varies considerably. Certain institutions, such as those in Hungary or Jordan, demonstrate excellent levels of training, as shown by their graduates' superior performance rates in Israeli certification examinations, while other institutions, mostly located in non-OECD countries, have generally failed to provide adequate medical training, resulting in inadequate medical competence among some of their graduates [28, 32, 33]. Consequently, the "Yatziv" reform was implemented to prevent graduates of those medical schools from obtaining an Israeli medical license. According to data from the Ministry of Health, 60% of the graduates of foreign medical schools who received a license to practice in 2022 had studied in medical schools that will no longer be considered for an Israeli license as of from 2025 [28, 32]. This will particularly impact Israel's remotely located medical centers. For example, 62% and 46% of the doctors in the Ziv Medical Center in Safad and the Galilee Medical Center in Nahariya (both located in northern Israel) studied in foreign medical schools that were excluded by the "Yatziv" reform, compared to only 9% of physicians in Tel-Aviv Medical Center, which is located in central Israel, studied in such schools [33].

The present study evaluated Israeli medical students' considerations during their last year of medical school with regard to choosing a residency program in remotely located medical institutions, and what incentives might influence their choice. The results of this study are especially important at present when the Ministries of Health and Finance together with the Israeli Medical Association are about to sign a new collective agreement, with the intention of helping to guide decision-making by those policymakers.

## Methods

This study included Israeli medical students who studied in Israeli universities and those who studied in foreign medical schools during their final year of medical education and before the start of their year of internship. We did not receive any official information regarding the details of the Israeli or foreign medical students. A "remotely located hospital" was defined according to the list of hospitals selected by the Israeli Ministry of Health in the 2011 physicians' collective agreement as eligible for the financial incentive program [27].

Students were recruited voluntarily between July and November 2022 via invitations distributed by Israeli faculties of medicine or through Facebook and WhatsApp groups. A total of 704 Israeli medical school graduates had participated in the internship lottery in January 2022 (a single lottery for local medical school graduates takes place during the last year of medical school). A total of 1936 Israeli students from foreign medical schools participated in 5 consecutive lotteries for foreign graduates that took place during 2023 (there are multiple lotteries for foreign medical students every year taking place upon completion of medical education) [34]. We used those numbers as estimators for the number of students in each group. Inclusion criteria were Israeli medical students of all ages, sex, and ethnicities who were in their final year of medical education in all faculties of medicine in Israel and abroad. Students who completed the questionnaire were entered into a lottery for a chance to win a laptop computer. The study was approved by the Sheba Medical Center institutional review board (SMC-22-9360) in accordance with the Declaration of Helsinki, and all participants provided informed consent in the questionnaire.

### Study questionnaire

We built an online questionnaire (supplementary material 1) previously validated by Israelis from different linguistic backgrounds (Hebrew, Russian, Arabic, English). The questionnaire included multiple-choice demographic questions and a 5-point Likert scale questionnaire on the effects of specific interventions on the decision to select a residency in a remotely located medical center on a scale ranging from 1 ("strongly disagree") to 5 ("strongly agree"). The questionnaire was concluded with the item "No incentive could influence me to start my residency in a remotely located institution."

### Statistical analysis

Categorical variables were described by frequency and percentage. Continuous variables were summarized by median and range. Chi-square or Fisher's exact test and the Mann–Whitney U test were used to study categorical and continuous variables, respectively. The Benjamini‒Hochberg procedure (false discovery rate) was used to adjust the *p*-values to avoid errors due to multiple comparisons. Results of the 5-point Likert scale results are usually considered as ordinal rather than continuous data [35]. Therefore, responses were clustered into two categories: "incentive receptive" (scores 4 and 5 representing agree and strongly agree) versus "non-receptive" (scores 1–3 representing strongly disagree, disagree and neutral). A univariable logistic regression model was created to study factors associated with incentive receptivity. The multivariable logistic regression model included variables meeting a *p*-value < 0.2 in the univariable analysis after correction for multiple comparisons. All *p*-values were two-sided, and *p-value* < 0.05 was considered statistically significant. Data were analyzed using R software (version 4.1.2).

## Results

### Study population

A total of 522 students responded to the questionnaire. The median age was 30 years (range 23–58), 52% were females, 86% were Jewish, and 13% were Arabs. There were 405 (78%) participants who studied in local medical schools (405/704 [58%] in the class of 2023 [34]), and 117 (22%) students who studied abroad (117/1936 [6%] of the foreign students who entered the internship lotteries in 2023). Most of the students were married or in a long-term relationship, and 70%, 23%, and 5% of the students had lived during their childhood in the central, northern, and southern Israeli regions, respectively, while 2% had been raised abroad. A total of 219 (42%) students had at least one medical clerkship in a remotely located hospital in Israel, and 120 (24%) students selected at least one such hospital among their top five choices in their list of preferences for their internship year (Table 1).

**Table 1:** Students' characteristics and demographics

Characteristic	n = 522
*Institution and program*	
Bar-Ilan University—3/4 year program	68 (13%)
Ben Gurion University—6-year program	54 (10%)
Hebrew University—6-year program	102 (20%)
Technion Institute—6-year program	63 (12%)
Tel Aviv University—4-year program	38 (7%)
Tel Aviv University—6-year program	80 (15%)
Foreign medical schools	117 (22%)
Gender: female	270 (52%)
Age, years (range)	30.0 (23–58)
*Religion*	
Jewish	448 (86%)
Arab	66 (13%)
Other	8 (2%)
*Family status*	
Single	190 (36%)
Married/long-term relationship	329 (63%)
Divorced	3 (1%)
Have children	129 (24%)
*Socioeconomic background*	
Below average	51 (10%)
Average	319 (62%)
Above average	143 (28%)
*Geographical region in childhood*	
Center	340 (70%)
North region	110 (23%)
South region	24 (5%)
Abroad	10 (2%)
*Geographical region of parents*	
Center	336 (70%)
North region	107 (22%)
South region	22 (5%)
Abroad	13 (3%)
*Geographical region at present*	
Center	327 (68%)
North region	113 (23%)
South region	37 (8%)
Abroad	6 (1%)
*Geographical region of spouse's parents*	
Center	235 (68%)
North region	78 (22%)
South region	16 (5%)
Abroad	19 (5%)
*Spouse's work type*	
Flexible or remote working option	166 (44%)
Not flexible	161 (42%)
Not working	23 (6%)
Other	35 (9%)
*Residency type*	
Surgical	171 (33%)
Non-surgical	266 (51%)
No residency desired	3 (1%)
Not yet decided	82 (16%)
Clerkship in remotely located institution during medical school	219 (42%)
*Remotely located institution was in top 5 choices for internship*	
Yes	120 (24%)
No	326 (64%)
Not yet participated	63 (12%)
*Specific department desired for residency*	
No	286 (55%)
Yes	229 (44%)
Personal preference of residency in remote area	69 (13%)

Only 69 (13%) students reported that they prefer to enroll in a residency program in a remotely located medical center (strongly agree, 4.5%; agree, 8.6%). Table 2 presents univariable and multivariable logistic regression analyses of factors associated with their choice. The factors that were significantly associated with that preference in the multivariable analysis were non-Jewish religion, currently residing in those areas, and parents residing in those areas. Notably, neither medical education nor a clerkship in remotely located institutions were associated with this preference in the multivariable analysis. In addition, 79% of the students responded that they believe the Soroka Medical Center in Be'er Sheva is different from the other medical centers listed as "remotely located" hospitals in the 2011 incentive programs, given that it is a university-affiliated tertiary center.

**Table 2:** Univariable and multivariable logistic regression analyses of factors associated with a preference for residency program in remotely located institution

Characteristic	OR (95% CI); p*	
	Univariable	Multivariable †
Gender: male	1.68 (1.01–2.83); 0.085	1.55 (0.84–2.90); 0.16
Age, years	1.01 (0.94–1.08); 0.80	
Israeli vs. foreign medical school	0.52 (0.30–0.92); 0.04	0.67 (0.30–1.56); 0.34
Medical education in remotely located university	1.79 (1.02–3.07); 0.07	1.81 (0.76–4.44); 0.19
Family status (single/divorced vs. married)	0.96 (0.56–1.62); 0.93	
Children (0 vs. ≥ 1)	0.78 (0.45–1.39); 0.48	
Religion (non-Jewish vs. Jewish)	5.10 (2.86–9.02); < 0.001	2.35 (1.02–5.34); 0.04
Socioeconomic background (average/below average vs. above average)	1.75 (0.95–3.44); 0.14	0.78 (0.37–1.68); 0.51
Dwelling in remote region at childhood (vs. center/abroad)	5.63 (3.22–10.0); < 0.001	1.33 (0.54–3.50); 0.54
Dwelling in remote region at present (vs. center/abroad)	7.81 (4.31–14.8); < 0.001	3.67 (1.66–8.47); 0.002
Dwelling in remote region of parents/spouse's parents at present (vs. center/abroad)	6.00 (3.33–11.3); < 0.001	2.85 (1.06–7.42); 0.03
Desired residency type (surgical vs. non-surgical)	1.02 (0.58–1.77); 0.93	
Clerkship in remotely located institution during medical school	2.26 (1.36–3.82); 0.01	0.96 (0.4–2.22); 0.93

* *P*-values were corrected for multiple comparisons using the Benjamini–Hochberg procedure (false discovery rate)

† Variables with *p* < 0.2 in the univariable model were introduced into the multivariable model

### Incentive responsiveness

**Fig. 1:**
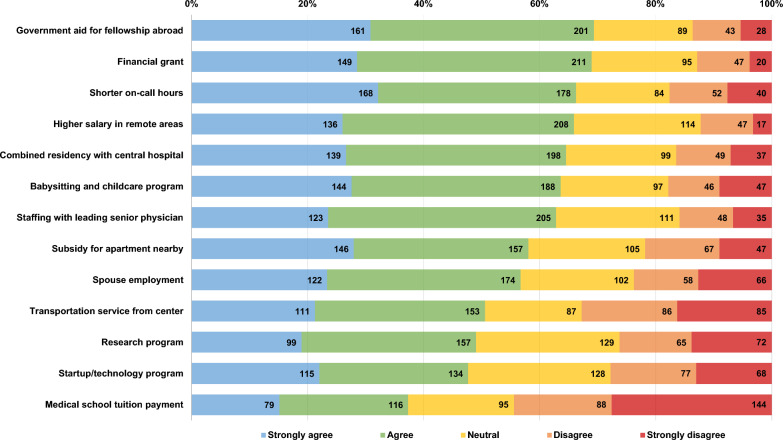
Results of 5-point Likert scale questionnaire evaluating Israeli medical students’ receptivity to different suggested incentives. Variables are ordered by their receptivity (the percentage of students who agree and strongly agree that the program could affect their decision). The number of students is listed in each box

The results of the 5-point Likert scale that evaluated different suggestions for incentive programs are presented in Fig. 1. They are given in order of their total receptivity (the percentage of students agreeing and strongly agreeing that it may positively influence them to choose a remotely located residency program). The most influential incentives noted by the students were: 1. government assistance in acceptance to and financial support for a future fellowship abroad; 2. a financial grant; and 3. fewer on-duty hours. The least influential incentives were a payment for medical school tuition and a startup/technology-combined residency program. However, it should be noted that most of the participating students were studying in universities in Israel, where the total medical school tuition is approximately 80K NIS for a 6-year program, significantly lower than the tuition of universities abroad. Notably, only 7% of the students responded that no incentive program would influence them to choose a residency in remotely located institutions. Moreover, students who reported a preference for them were not more receptive to incentives (*p*-value = 0.52).

Supplementary Table 1 presents differences in their receptivity to incentives between participants who studied medicine in Israel and those who studied abroad. There was no difference between the two groups regarding the percentage of the students who indicated that no incentive could influence their decision to choose a residency program in remotely located medical centers. However, students in the latter group were more receptive to direct financial incentives: medical school tuition payment, a higher salary, and government subsidy for the purchase of an apartment near the hospital.

Table 3 presents univariable and multivariable logistic regression models evaluating the association between various factors and incentive receptivity as reported by the students. Factors significantly associated with decreased responsiveness were male sex and older age. Surprisingly, current dwelling in a remotely located region was not associated with incentive receptivity in the multivariable model. We further explored the two most influential incentives reported by the students, government assistance in acceptance to and financing of a future fellowship abroad and a financial grant. Univariable and multivariable logistic regression analyses of factors associated with receptivity to government assistance for a fellowship abroad are presented in Supplementary Table 2, and the only factor that reached a level of significance was the preference for a surgical residency. Supplementary Table 3 presents the univariable and multivariable logistic regression analyses of factors associated with receptivity for a financial grant, and the only variable that reached a level of significance was the choice of a remotely located hospital for internship.

**Table 3:** Univariable and multivariable logistic regression analyses of factors associated with receptiveness to incentives

Characteristic	OR (95% CI); p*	
	Univariable	Multivariable †
Gender: male	0.55 (0.35–0.86); 0.03	0.42 (0.22–0.78); 0.007
Age, years	0.92 (0.86–0.97); 0.03	0.91 (0.83–0.98); 0.02
Israeli vs. foreign medical school	1.46 (0.87–2.39); 0.23	
Medical education in remotely located university	2.44 (1.33–4.85); 0.03	3.42 (1.29–9.63); 0.02
Family status (single/divorced vs. married)	0.92 (0.59–1.46); 0.79	
Children (0 vs. ≥ 1)	1.09 (0.65–1.79); 0.79	
Religion (non-Jewish vs. Jewish)	0.96 (0.52–1.86); 0.90	
Socioeconomical background (average/below average vs. above average)	1.64 (1.02–2.63); 0.12	1.72 (0.92–3.20); 0.09
Dwelling in remote region at childhood (vs. center/abroad)	1.23 (0.73–2.12); 0.56	
Dwelling in remote region at present (vs. center/abroad)	1.64 (0.98–2.85); 0.14	1.28 (0.54–3.15); 0.58
Dwelling in remote region of parents/spouse's parents at present (vs. center/abroad)	1.33 (0.82–2.20); 0.35	
Desired residency type (surgical vs. non-surgical)	0.66 (0.40–1.08); 0.18	0.78 (0.42–1.46); 0.44
Clerkship in remotely located institution during medical school	1.56 (0.99–2.52); 0.14	0.55 (0.24–1.3); 0.16
Remotely located hospital in first 5 choices for internship	3.56 (1.81–7.86); 0.01	3.46 (1.37–10.6); 0.01

* *P*-values were corrected for multiple comparisons using the Benjamini–Hochberg procedure (false discovery rate)

† Variables with *p* < 0.2 in the univariable model were introduced into the multivariable model

## Discussion

In the former Israeli Medical Association collective agreement with the major physicians' employers (government and health maintenance organizations [HMOs]), almost 1 billion NIS was invested to attract young physicians to residency programs in remotely located institutions. A new agreement is imminent, and we aimed to gauge the effectiveness of different possible incentives to attract residents to those hospitals. The main finding of this study was that only 7% of the students stated that no intervention could influence them to select a remotely located residency program. This finding indicates that selection of the right interventions may very well achieve the goal of increasing the number of students who select those residency programs.

The incentive selected by the students as most influential in their decision to choose a remotely located residency program was government assistance in finding and funding a future fellowship program abroad. This incentive was more influential among students who reported a preference for surgical residency. This finding aligns with the reports in the literature that prestige and career prospects are often more significant factors for students pursuing surgical residencies than family or residency location [16–19]. McClintock et al. showed that pursuing fellowship training was associated with the desire to have an academic career, being involved in teaching, and hold leadership positions among general surgery residents [36]. Such incentives could benefit remotely located institutions in two ways: first, by returning physicians who had been skilled abroad to their remotely located institutions, and second, by fulfilling students' aspirations for personal development by providing fellowships abroad. Other incentives that were noted by students as being influential were financial grants and the shortening of on-call shifts, a topic that is currently under debate in Israel. In September 2023, on-call hours were shortened only in remotely located hospitals and in specific fields (internal medicine, geriatrics and emergency medicine) from 26 to 21 h. The effect of this intervention has not yet been studied.

Similar to previous reports, present dwelling and the presence of a parent were associated with a preference for a residency program in remotely located institutions [1, 37–40]. It is noteworthy that neither studying in a university located in a remote area nor performing a medical clerkship in a remotely located hospital was independently associated in the multivariable analysis with the preference for completing a residency program in those locations, as also reported by others [9, 37, 40]. This might indicate that of those who studied in the periphery, the preference for that location lies on their baseline characteristics rather than on the actual peripheral exposure during medical school. Nonetheless, in the multivariable logistic regression that examined the characteristics of students who were receptive to incentives revealed that medical education in a remotely located university was indeed significantly associated with incentive responsivity as an independent factor, although a single clerkship was not. This suggests that prolonged exposure to remotely located hospitals through medical education may increase students' willingness to enroll in their residency programs if appropriate incentives are provided, whereas a brief clerkship may not have the same effect. One possible explanation is that students who complete their entire education at a peripheral university often move to and become integrated into the surrounding communities, gaining exposure to local opportunities, culture, and mindset. This level of immersion is unlikely to occur during a short clerkship.

Furthermore, this study revealed differences between medical students who studied medicine abroad and those who studied locally. The former group was more receptive to financial incentives, such as governmental subsidies for purchasing an apartment near the hospital, higher salaries, and government reimbursement for medical school tuition. Tuition reimbursement and educational loans are used in some countries as an incentive to attract students to work in remote areas [4]. Such incentives may be particularly attractive to Israeli students who studied medicine abroad and who had borne the higher tuition costs compared to Israeli schools. Understanding the desires and needs of Israeli medical students who study abroad is crucial for effective intervention given that more than one-half of the newly licensed physicians in Israel in 2022 were graduates of foreign medical schools.

Research or startup programs in remotely located hospitals were found attractive by some participants, however, not as attractive as the financial incentive. Specifically, while 69% and 68% of the students were receptive to government aid for a fellowship abroad and to financial grants, respectively, only 49% and 47% were receptive to research and startup programs in peripheral hospitals, respectively. Furthermore, although the program of establishing centers of excellence by staffing leading physicians from abroad or from centrally located hospitals was not chosen by the students as one of the most influential incentives, it is important to consider the potential benefits of such an approach to assist in the process of applying for fellowships abroad through their connections and influence and their adding prestige to the teaching staff.

Most of students in our cohort distinguished between Soroka Medical Center and other hospitals designated as "remotely located" in the 2011 agreement, primarily because it is the only remotely located university-affiliated tertiary medical center that participated in the incentive program. It is currently the sole hospital in the Negev area, a semi-desert region in southern Israel covering about 60% of the country's territory. Although located in the heart of Be'er Sheva, the district's capital, Soroka was ranked only between the 7th and 11th place in students' preferences for internship placements among Israeli medical school graduates between 2022 and 2024 [34]. This ranking highlights the challenges Soroka faces in attracting medical students compared to tertiary centers in central Israel. The hospital's inclusion in the 2011 incentive program was part of a government effort to strengthen the Negev area and specifically Soroka, as was also recommended by the "Afek" committee [41]. The difficulties in recruiting interns to this hospital suggest that staffing challenges in Israel's remote areas are influenced not only by technological or professional factors but also by geographic location. As noted earlier, the findings of our study as well as those of others [3, 42] indicate that students with personal or parental ties to remote areas are more likely to prefer residency in those areas. Therefore, we believe that enhancing and initiating programs like the "Ilanot" program, which targets and supports students with "significant ties" to remote regions, could help recruit students who might eventually remain and practice there. However, the preference of medical students originally from remote regions could face legal challenges.

This study has several limitations. Some argue that incentives that are based exclusively upon the choice of residency location may potentially allocate resources to physicians who would have made the same decision without them [8]. Furthermore, the concern of bias is common in this type of research. Those planning a residency in the remote regions might not want to forgo the opportunity to receive a grant, thus introducing confirmation bias [8, 27]. However, this study shows that students who maintained a preference for residency in those regions were not more receptive to incentive programs. Furthermore, factors, such as present personal or parental dwelling in the periphery, and geographical region in childhood—factors that are usually associated with the choice of periphery practice [6, 8, 43]—were not associated with incentive receptivity in the current study. In addition, because Israel is relatively small in size, it is somewhat difficult to compare its remote or rural areas with those in larger countries, such as Australia, or Canada. However, we believe the public perception of these areas is similar, and that medical staffing challenges are common to them all. Therefore, it is possible to draw relevant conclusions from the Israeli experience to other countries. Additionally, this study did not differentiate between hospitals based on their distance from the center, whether they are located in 'mid' or 'far' regions. Future research is needed to assess whether students perceive these hospitals differently. Finally, conclusions regarding the way incentives may or may not affect decisions of students graduating abroad are limited by the low percentage of participants from this subgroup. Further research involving this group is warranted, especially given that this study showed group differences in incentive receptivity. A strength of this report is the relatively high number of students who participated, reflecting almost 60% of the graduates from Israeli medical schools in 2023.

## Policy implication

Based upon the findings of this study, we recommend that several key considerations should be addressed in the negotiations between the Ministry of Health, the Ministry of Finance, and the Israel Medical Association in the new physician agreement. Financial grants and increasing staffing ratios alone are insufficient, while innovative incentive programs are pivotal and should be tailored according to the region and hospital's needs. The latter could include government support in registration, admission, and financing of fellowships abroad, as well as staffing remotely located hospitals with leading physicians. Moreover, it is important to strengthen programs that target and cultivate students and youth with close ties to peripheral regions as the next generation of physicians and by providing preferential admission allocations for them in medical schools. Additionally, comprehensive programs that consider the needs of residents' families, such as childcare, spouse employment, and housing subsidies near hospitals, are needed. This study also highlights the necessity of tailoring incentives to different populations, since preferences differ between graduates of Israeli and foreign medical schools. Finally, providing combined residency and sub-specialty programs with central tertiary medical centers should also be considered.

## Conclusion

This study reports the attitude of today’s Israeli medical school graduates toward various elements of incentive programs aimed at attracting them to a residency in remote locations. Government intervention should be tailored to fit these graduates' needs and preferences in order to be more influential and cost-effective. Additional studies are needed to evaluate the preferences of Israeli medical students attending foreign institutions, given their underrepresentation in this study. Moreover, future research will be needed to compare the results of the current study with the students’ ultimate decisions once the new incentive programs are in place.

### Supplementary Information

Additional file 1,

Additional file 2,

## Data Availability

The datasets used and/or analyzed during the current study are available from the corresponding author on reasonable request.

## Abbreviations

NIS: New Israeli Shekel
OECD: Organization for economic co-operation and development

## References

[1] Wilson NW, Couper ID, De Vries E, Reid S, Fish T, Marais BJ. A critical review of interventions to redress the inequitable distribution of healthcare professionals to rural and remote areas. Rural Remote Health. 2009;9:1060. 19530891

[2] Ono T, Schoenstein M, Buchan J. Geographic Imbalances in Doctor Supply and Policy Responses. OECD Heal Work Pap. Paris; 2014;No. 69.

[3] Feder-Bubis P, Bin-Nun G, Zarhin D, Sherf M, Heiman-Neuman N. Residents’ choice of a placement in periphery hospitals in Israel: the significance of personal/family and professional considerations. Health Policy. 2023;132:104795. 36990021 10.1016/j.healthpol.2023.104795

[4] Dussault G, Franceschini MC. Not enough there, too many here: understanding geographical imbalances in the distribution of the health workforce. Hum Resour Health BioMed Central. 2006;4:1–16. 10.1186/1478-4491-4-12 PMC1481612 16729892

[5] Lehmann U, Dieleman M, Martineau T. Staffing remote rural areas in middle- and low-income countries: a literature review of attraction and retention. BMC Health Serv Res BioMed Central. 2008;8:1–10. 10.1186/1472-6963-8-19 PMC2259330 18215313

[6] Ashkenazi Y, Gordon M, Rosen B. Using financial incentives to attract medical residents to the periphery: the Israeli experience. Health Policy. 2019;123:80–6. 30340905 10.1016/j.healthpol.2018.10.006

[7] Holte JH, Kjaer T, Abelsen B, Olsen JA. The impact of pecuniary and non-pecuniary incentives for attracting young doctors to rural general practice. Soc Sci Med Pergamon. 2015;128:1–9. 10.1016/j.socscimed.2014.12.022 25569609

[8] Wasserstrum Y, Magnezi R, Tamir O, Koren S, Lotan D, Afek A. Self-reported influence of monetary grants in the choice of a medical residency in remote or under-served areas. Isr J Health Policy Res. 2019;8:1–9. 30764867 10.1186/s13584-018-0272-6 PMC6376660

[9] DeMiglio L, Jolicoeur J, Lamb IR, Cousins M, Nutbrown L, Orrantia E. Draw to Practice: A Qualitative Study Examining Factors Attracting Physicians to Rural Northern Ontario. Cureus [Internet]. Cureus Inc.; 2024 [cited 2024 Jun 15];16. Available from: /pmc/articles/PMC10977940/10.7759/cureus.55074 PMC10977940 38550479

[10] Arredondo K, Touchett HN, Khan S, Vincenti M, Watts B V. Current Programs and Incentives to Overcome Rural Physician Shortages in the United States: A Narrative Review. J Gen Intern Med [Internet]. Springer; 2023 [cited 2024 Jun 15];38:916. Available from: /pmc/articles/PMC10356718/10.1007/s11606-023-08122-6 PMC10356718 37340266

[11] Brütting C, Herget S, Nafziger M, Klingenberg A, Deutsch T, Frese T, et al. Factors promoting willingness to practice medicine in rural regions and awareness of rural regions in the university’s catchment area – cross-sectional survey among medical students in central Germany. GMS J Med Educ [Internet]. German Medical Science; 2023 [cited 2024 Jun 15];40. Available from: /pmc/articles/PMC10407585/10.3205/zma001634 PMC10407585 37560039

[12] Schroeder H, Shacham A, Amar S, Weissman C, Schroeder JE. Comparison of medical students’ considerations in choosing a specialty: 2020 vs. 2009/10. Hum Resour Health. BioMed Central Ltd; 2024;22:1–9. 10.1186/s12960-023-00885-7 PMC10773044 38191435

[13] Cribari M, Holzer BM, Battegay E, Minder CE, Zimmerli LU. What makes internal medicine attractive for the millennial generation? A survey of residents in internal medicine in Switzerland. Swiss Med Wkly NLM (Medline). 2018;148:w14696–w14696. 10.4414/smw.2018.14696 30552857

[14] Kleinert R, Fuchs C, Romotzky V, Knepper L, Wasilewski ML, Schröder W, et al. Generation Y and surgical residency – passing the baton or the end of the world as we know it? Results from a survey among medical students in Germany. PLoS One. 2017;12:e0188114. 29176812 10.1371/journal.pone.0188114 PMC5703530

[15] Mazeh H, Mizrahi I, Eid A, Freund HR, Allweis TM. Medical students and general surgery—Israel’s national survey: lifestyle is not the sole issue. J Surg Educ. 2010;67:303–8. 21035770 10.1016/j.jsurg.2010.07.015

[16] Baschera D, O’Donnell Taylor E, Masilonyane-Jones T, Isenegger P, Zellweger R. Are medical students who want to become surgeons different? An international cross-sectional study. World J Surg. 2015;39:2908–18. 26296833 10.1007/s00268-015-3195-1

[17] Azizzadeh A, McCollum CH, Miller CC, Holliday KM, Shilstone HC, Lucci A. Factors influencing career choice among medical students interested in surgery. Curr Surg. 2003;60:210–3. 14972298 10.1016/S0149-7944(02)00679-7

[18] Kuzukiran Y, Kurtul B, Kurtul B, Tarhan Z, Tas O, Azap A. The factors affecting specialty preference and job satisfaction of medical residents. Infect Dis Clin Microbiol. 2019;1:42–6. 10.5152/idcm.2019.19006

[19] Ladha FA, Pettinato AM, Perrin AE. Medical student residency preferences and motivational factors: a longitudinal, single-institution perspective. BMC Med Educ. 2022;22:187. 35300656 10.1186/s12909-022-03244-7 PMC8929265

[20] Grasreiner D, Dahmen U, Settmacher U. Specialty preferences and influencing factors: a repeated cross-sectional survey of first- to sixth-year medical students in Jena, Germany. BMC Med Educ. 2018;18:1–11. 29743057 10.1186/s12909-018-1200-8 PMC5944057

[21] Ashkenazi Y, Gordon M, Yankelevitz A, Rosen B. Attracting medical residents to rural areas and medical fields in severe physician shortages following the 2011 collective agreement. 2017.

[22] Defining localities and areas as having national priority: State of Israel governmental desicion no. 1060 [Internet]. 2009 [cited 2024 Aug 10]. Available from: https://www.gov.il/he/pages/2009_des1060

[23] The workforce in the health professions‬ (Hebrew) [Internet]. Jerusalem; 2022 Nov. Available from: https://www.gov.il/BlobFolder/reports/health-professions-manpower/he/files_publications_units_info_manpower2021.pdf

[24] Jacobson E, Ezra V. Financial incentives as a governmental tool to bridge the medical manpower gap between Israel’s center and periphery. Isr J Health Policy Res. 2019;8:1–4. 31610814 10.1186/s13584-019-0344-2 PMC6791004

[25] Blank R. The effectiveness of the grant program to encourage the work and specialization of doctors in the periphery [Internet]. 2024 Jan. Available from: https://www.gov.il/BlobFolder/reports/effectiveness-grant-program-encourage-work-specialization-doctors-periphery-2011-2019/he/files_publications_units_financial-strategic-planning_publications_man-power_2_14de7f6e-1cef-ed11-815b-005056aa4246_11_20418.pdf

[26] Notice regarding the end of the grants to doctors in professions in need in the center and the periphery, and professions with a staffing shortage in the periphery (Hebrew) [Internet]. 2019 [cited 2023 Apr 21]. Available from: https://www.ima.org.il/userfiles/image/maanakim2020.pdf

[27] Israeli Central Bureau of Statistics. Examining the effectiveness of the doctors agreement on specializing in the periphery and professions in need (Hebrew) [Internet]. 2020. Available from: https://www.gov.il/BlobFolder/guide/research_and_data/he/research-and-data_files_research-file6.pdf

[28] Rachel Brenner-Shalem, Alexey Belinsky, Jonathan Uziely, Naama Yona, Ayelet Grinbaum Arizon. The reform in medical manpower: Ministry of Health policy in the field of the shortage of doctors in Israel and the empowerment of the Negev and the Galilee (Hebrew) [Internet]. Jerusalem; 2023 Jan. Available from: https://www.gov.il/BlobFolder/reports/health-human-resources-reform-n/he/files_publications_units_financial-strategic-planning_publications_man-power_health-human-resources-reform.pdf

[29] Ilanot program [Internet]. [cited 2024 Apr 13]. Available from: https://ilanot-program.co.il/

[30] Weissman C, Zisk-Rony RY, Avidan A, Elchalal U, Tandeter H. Challenges to the Israeli healthcare system: attracting medical students to primary care and to the periphery. Isr J Health Policy Res. 2018;7:1–17. 29843802 10.1186/s13584-018-0218-z PMC5975704

[31] OECD Report on Medical Education and Training in Israel [Internet]. 2023 May. Available from: https://www.oecd.org/health/OECD-report-on-medical-education-and-training-in-Israel.pdf

[32] Angel Y, Fire G. Healthcare policy changes in an era of health workforce shortage. Isr J Health Policy Res. 2023;12:1–6. 37563656 10.1186/s13584-023-00576-7 PMC10413592

[33] Brenner-Shalem R, Alexey Belinsky. Long-term planning of doctors in Israel [Internet]. 2022. Available from: https://www.gov.il/BlobFolder/reports/manpower-planing-15122021-n/he/files_publications_units_financial-strategic-planning_publications_man-power_manpower-planing-15122021.pdf

[34] Israeli Ministry of Health, Medical Internship Raffle Data (Hebrew) [Internet]. 2024 [cited 2024 Mar 23]. Available from: https://www.gov.il/he/departments/general/medical-internship-raffle

[35] Sullivan GM, Anthony R. Artino J. Analyzing and Interpreting Data From Likert-Type Scales. J Grad Med Educ. Accreditation Council for Graduate Medical Education; 2013;5:541. 10.4300/JGME-5-4-18 PMC3886444 24454995

[36] McClintock NC, Gray KE, Neville AL, Kaji AH, Wolfe MM, Calhoun KE, et al. Factors associated with general surgery residents’ decisions regarding fellowship and subspecialty stratified by burnout and quality of life. Am J Surg. 2019;218:1090–5. 31421896 10.1016/j.amjsurg.2019.08.003

[37] Easterbrook M, Godwin M, Wilson R, Hodgetts G, Brown G, Pong R, et al. Rural background and clinical rural rotations during medical training: effect on practice location. C Can Med Assoc J. 1999;160:1159. PMC1230268 10234346

[38] McGrail MR, Humphreys JS, Joyce CM. Nature of association between rural background and practice location: A comparison of general practitioners and specialists. BMC Health Serv Res BioMed Central. 2011;11:1–8. 10.1186/1472-6963-11-63 PMC3074548 21429224

[39] Rabinowitz HK, Diamond JJ, Markham FW, Paynter NP. Critical factors for designing programs to increase the supply and retention of rural primary care physicians. JAMA Am Med Assoc. 2001;286:1041–8. 10.1001/jama.286.9.1041 11559288

[40] Woloschuk W, Tarrant M. Does a rural educational experience influence students’ likelihood of rural practice? Impact of student background and gender. Med Educ. 2002;36:241–7. 11879514 10.1046/j.1365-2923.2002.01143.x

[41] The Committee for Examining the Expansion of Medical Services in the South: A Summary Report to the Minister of Health and the Director General of the Office of the Prime Minister [Internet]. 2014 Aug. Available from: https://www.health.gov.il/publicationsfiles/darom.pdf

[42] Treister-Goltzman Y, Peleg R. The physician shortage in Israel and a policy proposal for improvement. Isr J Health Policy Res. 2023;12:1–11. 36793111 10.1186/s13584-023-00552-1 PMC9931442

[43] Tate RB, Aoki FY. Rural practice and the personal and educational characteristics of medical students: Survey of 1269 graduates of the University of Manitoba. Can Fam Phys. 2012;58:e641. PMC3498038 23152471

