# Supplementary material for: Attracting medical school graduates to residency programs in remotely located hospitals: the challenge lies beyond financial incentives
Source: Isr J Health Policy Res. 2024 Aug 26;13:40. doi: 10.1186/s13584-024-00629-5 (PMC11346137; doi:10.1186/s13584-024-00629-5)
Supplement: Supplementary file 1 — Additional file 1, [file 13584_2024_629_MOESM1_ESM.docx]

**Supplementary material 1.** Study questionnaire (translated from Hebrew)

Before the renewal of the physicians' collective agreement, we would like to study and influence the policy regarding governmental encouragement to start residency programs in remotely located hospitals. Therefore, we are conducting a study among Israeli medical students in Israel and abroad during their final year of medical school, intending to examine which of the following incentives may influence to choose a residency program remotely located hospital.

This questionnaire is anonymous unless you choose to share your personal information at the beginning. Among those who fill in their details, 5 tablet computers will be drawn.

Duration of answering the questionnaire: about 3-5 minutes.

* The questionnaire is worded in general language (Hebrew) and is aimed at both men and women.

**I agree to participate in the study (yes/no).**

**I agree to fill in the personal details (yes/no).**

**Personal Information-**

- Name -
- Email-
- Phone -

**Academic institution-**

- Ariel University
- Ben-Gurion University
- Bar Ilan University - Safed - four-year program
- Bar Ilan University - Safed - three-year program
- Tel Aviv University - four-year program
- Tel Aviv University - six-year program
- The Hebrew University
- Haifa Technion
- Ariel University

**Year of Birth:**

**Gender:**

**Family Status:**

**Economic background:**

- Below average
- Average
- Above average

**Number of children:**

**Population group and religion:**

- Jews
- Arabs: Muslims (including Circassians), Christian-Arabs (including Armenians), Bedouin and Druze.
- Other: non-Arab Christians, members of other religions and without religious classification.

**Country of birth:**

**City of residence during high school:**

**Where do your parents currently live?**

**Current city of residence**:

**Spouse's city of residence during high school:**

**Where do your partner's parents live?**

**Characteristics of the spouse's workplace**

- - In-Office, no remote work option
  - In-Office, but can work remotely
  - Can work remotely
  - Spouse does not work
  - Other

**At this stage, in which medical field would you like to specialize?**

- Oncology
- Pathology
- Public health
- Geriatrics
- Anesthesia
- Obstetrics and gynecology
- Urology
- Orthopedics
- General Surgery
- Plastic surgery
- Cardiothoracic surgery
- Pediatric surgery
- Vascular surgery
- Otorhinolaryngology
- Dermatology
- Ophthalmology
- Clinical Microbiology
- Clinical laboratory medicine
- Neurosurgery
- Neurology
- Psychiatry (child/adult)
- Radiology
- Nuclear medicine
- Emergency Medicine
- Forensic medicine
- Physical medicine and rehabilitation
- Internal medicine
- Occupational medicine
- Pediatrics
- Family medicine
- I do not want to specialize after the internship
- still do not know

**Is there a specific department in which you would like to specialize?**

*** For the purpose of this study, "****remotely located hospitals"*** *are those defined according to the grant program introduced by the Ministry of Health: Ha'emek in Afula, Soroka in Be'er Sheva, the Western Galilee Hospital in Nahariya, Kiryat Shemona Health Center, the Center for mental health in Be'er Sheva, Ziv in Safed, Poriya Medical Cente in Tiberias, Barzilai in Ashkelon, Yoseftal Medical Center in Eilat, Mazra Mental Health Center in Mazra, Baka El Garbiya Health Center, Lev Hasharon Mental Health Center, Pardes Hanna Geriatric Center, Scottish Hospital in Nazareth, The French Hospital in Nazareth, The Holy Family Hospital Nazareth ***

**Do you intend to specialize in a remotely located institution?**

1. Certainly not
2. Apparently not
3. Neutral
4. Apparently yes
5. Certainly yes

**A remotely located institution was in the top five preferences I ranked in the internship ruffle -**

- Yes
- No

**In the following questions, you will be required to rate various incentives based on their degree of influence on your decision to choose a residency program in a remotely located hospital.**

**The rating is between 1-5, when:**

1. *Strongly do not agree (will have no influence at all)*
2. *Do not agree (will not influence)*
3. *Neutral*
4. *Agree (might influence)*
5. *Strongly agree (Will have a great impact, if this incentive is offered, I might decide to specialize in the periphery)*

**Incentives:**

- Financial grant (300-500 thousand NIS).
- Higher salary in remotely located institutions.
- Retrospective financing of the university tuition fee for MD degree.
- Combined Residency with a tertiary medical center in the center of the country.
- Governmental aid in funding and assistance in acceptance to a fellowship program abroad.
- Shortening of duty hours in remotely located hospitals.
- Transferring a department head or renowned senior physicians to remotely located department of residency from hospitals in the center of the country/abroad.
- Option to purchase an apartment at a subsidized price near the hospital.
- Finding a job for your spouse near the hospital.
- Kindergartens and babysitting programs in the hospital.
- Integrated research program.
- Integrated entrepreneurship/startup residency.
- Transportation to the hospital and back to my place of residence (so that I will not need to drive)
- No incentive can influence me to start residency in a remote area.

**In your opinion, is Soroka, being a tertiary center, largely different from the other remotely located hospitals?**

**Would you prefer to specialize in a large hospital?**
