# Supplementary material for: Attracting medical school graduates to residency programs in remotely located hospitals: the challenge lies beyond financial incentives
Source: Isr J Health Policy Res. 2024 Aug 26;13:40. doi: 10.1186/s13584-024-00629-5 (PMC11346137; doi:10.1186/s13584-024-00629-5)
Supplement: Supplementary file 2 — Additional file 2, [file 13584_2024_629_MOESM2_ESM.docx]

**Supplementary Table 1** Differences among Israeli and foreign medical students in receptiveness to incentives

| **Characteristic** | **Abroad**  n = 117 | **Israeli institution**  n = 405 | ***P*-value*** |  |
| --- | --- | --- | --- | --- |
| **Any influence of incentive** |  |  |  |  |
| An incentive might influence  No incentive might influence | 90 (77%)  27 (23%) | 336 (83%)  69 (17%) | 0.17 |  |
| **Financial grant** | 91 (78%) | 269 (66%) | 0.054 |  |
| **Higher salary** | 93 (79%) | 251 (62%) | 0.002 |  |
| **Payment for MD tuition** | 83 (71%) | 112 (28%) | <0.001 |  |
| **Combined residency with a central tertiary center** | 77 (66%) | 260 (64%) | 0.75 |  |
| **Governmental aid for fellowship abroad** | 88 (75%) | 274 (68%) | 0.17 |  |
| **Shorter night shifts in remotely located institution** | 88 (75%) | 258 (64%) | 0.054 |  |
| **Department head from abroad/central region** | 67 (57%) | 261 (64%) | 0.18 |  |
| **Subsidy for an apartment purchase near hospital** | 94 (80%) | 209 (52%) | <0.001 |  |
| **Employment for spouse near hospital** | 75 (64%) | 221 (55%) | 0.11 |  |
| **Babysitting/kindergarten near hospital** | 80 (68%) | 252 (62%) | 0.24 |  |
| **Combined residency and research program** | 66 (56%) | 190 (47%) | 0.11 |  |
| **Combined residency and startup program** | 65 (56%) | 184 (45%) | 0.11 |  |
| **Transportation from central region** | 70 (60%) | 194 (48%) | 0.054 |  |
| * *P*-values were corrected for multiple comparisons using the Benjamini–Hochberg procedure (false discovery rate). | | | | |

**Supplementary Table 2** Univariable and multivariable logistic regression analyses of factors associated with government assistance with fellowship abroad

|  | OR (95% CI); *p** | |
| --- | --- | --- |
| Characteristic | **Univariable** | **Multivariable** † |
| Gender: male | 0.87 (0.60-1.27); 0.51 |  |
| Age, years | 1.00 (0.95-1.06); 0.91 |  |
| Israeli vs. foreign medical school | 0.69 (0.43-1.09); 0.26 |  |
| Medical education in remotely located institution | 1.40 (0.89-2.24); 0.29 |  |
| Family status (single/divorced vs. married) | 1.27 (0.86-1.89); 0.34 |  |
| Children (0 vs. ≥1) | 1.24 (0.81-1.88); 0.38 |  |
| Religion (non-Jewish vs. Jewish) | 1.76 (0.98-3.18); 0.21 |  |
| Socioeconomic background (average/below average vs. above average) | 1.30 (0.86-1.96); 0.34 |  |
| Dwelling in remote region at childhood (vs. center/abroad) | 1.25 (0.81-1.96); 0.38 |  |
| Dwelling in remote region at present (vs. center/abroad) | 1.62 (1.05-2.55); 0.12 | 1.58 (0.89-2.88); 0.13 |
| Dwelling in remote region of parents/spouse's parents at present (vs. center/abroad) | 1.25 (0.83-1.89); 0.38 |  |
| Desired residency type (surgical vs. non-surgical) | 2.90 (1.83-4.70); <0.001 | 3.28 (1.92-5.79); <0.001 |
| Clerkship in remotely located institution during medical school | 1.36 (0.93-1.99); 0.26 |  |
| Remotely located hospital in top 5 choices for internship | 2.02 (1.24-3.36); 0.04 | 1.81 (0.99-3.42); 0.06 |

* *P*-values were corrected for multiple comparisons using the Benjamini–Hochberg procedure (false discovery rate)

† Variables with *p* < 0.2 in the univariable model were introduced into the multivariable model.

**Supplementary Table 3** Univariable and multivariable logistic regression analyses of factors associated with financial grant receptiveness

|  | OR (95% CI); *p** | |
| --- | --- | --- |
| Characteristic | **Univariable** | **Multivariable** † |
| Gender: male | 0.87 (0.60-1.27); 0.51 |  |
| Age, years | 0.97 (0.93-1.03); 0.35 |  |
| Israeli vs. foreign medical school | 0.57 (0.34-0.90); 0.04 | 0.69 (0.30-1.51); 0.36 |
| Medical education in remotely located university | 1.43 (0.91-2.29); 0.19 | 1.17 (0.53-2.58); 0.69 |
| Family status (single/divorced vs. married) | 1.31 (0.89-1.94); 0.24 |  |
| Children (0 vs. ≥1) | 1.32 (0.87-2.01); 0.24 |  |
| Religion (non-Jewish vs. Jewish) | 0.60 (0.92-2.93); 0.18 | 0.65 (0.29-1.50); 0.31 |
| Socioeconomic background (average/below average vs. above average) | 2.01 (1.34-3.01); 0.004 | 1.60 (0.95-2.69); 0.08 |
| Dwelling in remote region at childhood (vs. center/abroad) | 2.08 (1.31-3.37); 0.01 | 1.79 (0.65-4.81); 0.25 |
| Dwelling in remote region at present (vs. center/abroad) | 2.17 (1.39-3.44); 0.004 | 1.21 (0.60-2.48); 0.59 |
| Dwelling in remote region of parents/spouse's parents at present (vs. center/abroad) | 1.81 (1.20-2.77); 0.02 | 1.14 (0.48-2.88); 0.77 |
| Desired residency type (surgical vs. non-surgical) | 0.68 (0.45-1.03); 0.12 | 0.61 (0.37-1.01); 0.05 |
| Clerkship in remotely located institution during medical school | 1.69 (1.15-2.50); 0.02 | 1.08 (0.53-2.25); 0.84 |
| Remotely located hospital in top 5 choices for internship | 4.96 (2.80-9.39); <0.001 | 3.99 (2.03-8.51); <0.001 |

* *P-*values were corrected for multiple comparisons using the Benjamini–Hochberg procedure (false discovery rate).

† Variables with *p* < 0.2 in the univariable model were introduced into the multivariable model.
